# Milk Poisoning

**Published:** 1890-02-08

**Authors:** 


### HYGIENE.

### MILK POISONING.

Some of our readers may remember how, many years ago, a case of acute poisoning by a cheese, which happened to be of American manufacture, caused a scare that at the time threatened to create a prejudice against the imported article. It was supposed to be due to the presence of some fungus or mould, but in a subsequent case of the same kind, which occurred in America, Professor Vaughan was able to detect and isolate an alkaloid, to which he gave the name of Tyrotoxine, or cheese poison, and which was found to produce all the symptoms in question—purging, vomiting, convulsions, delirium, collapse, and coma. The origin of the poison itself remained unknown until an outbreak occurred in a fashionable American Hotel affecting all who had partaken of the evening's milk, those who had taken only the morning's milk escaping, though both were supplied from the same farm. Professor Vaughan discovered his Tyrotoxine, or, as it now appeared it would have been more correctly called, Galactotoxine, in the evening, but not in the morning, meal, and further inquiry elicited the fact that the cows were milked at the unusual hours of noon and midnight, or thereabouts, and that the midday milk had been immediately carted for eight miles on a rough road in the hottest hours of an unprecedentedly hot summer.

It is a fact well known to dairy farmers, at least in this country, that milk, if it is to be transported to any distance, as by rail, must be chilled, or, as the men say "killed," for if not it turns rapidly, and, indeed, acquires an unwholesomeness which cannot be entirely accounted for by the mere souring. That is to say, tyrotoxine is formed together with the lactic acid, and consequently all the great milk contractors and dairy companies make it a condition of their agreements with the farmers that the milk shall be passed through a refrigerator within a few minutes of drawing.

In the *Medical News*, Dr. Martin reports a case of a lady who, having breakfasted on bread and milk and coffee with milk, was immediately seized with convulsive movements of the limbs, a sensation of choking, and such weakness that she had to be carried to her bed. Five or ten minutes later she became delirious, and in less than half-an-hour totally unconscious, in which state she remained for about fifteen minutes. Convulsions continued after the return of consciousness for at least half-an-hour, the choking sensation still longer, while the headache and pain in the chest increased. An emetic of sulphate of zinc gave some relief, but she remained prostrate all the day, and did not regain her strength for a week. Her husband, who took the milk only with his coffee, felt merely faint and giddy, and was compelled to lie down for some time. Tyrotoxine was detected in the milk. Dr. Camman, in the *New York Medical Journal*, reports a similar illness which attacked 33 children in a school of 150, presumably from the same cause, though analysis of the milk gave a negative result, perhaps from the particular milk not having been examined.

We may here remark that our London patients who judge of the "richness" of milk by the rapidity with which the cream rises and its depth, frequently tell us that the milk which at our recommendation they have got from the Aylesbury, Welford's, the Belgravia, or other company, is not so rich and good as that they previously had from some neighbouring dairy; the fact being that the latter had not been chilled and early began to undergo decomposition: whereas chilled milk remains quite unchanged for at least twenty-four hours, and the cream rises very slowly, though a "separator" would in a few minutes show that the percentage of fat was quite as great. In fact, the properly-chilled country milk sent round in the morning, which will keep good till the evening in a suitable place, was milked in Wiltshire or Derbyshire at four or five p.m. on the previous day; whereas London *unchilled* milk would by that time have become totally unfit for use; and when brewer's grains form a part of the food of the cows, as they do largely in London and suburban dairies, the milk will often spoil within a few hours. Grains and ensilage are, we may add, strictly forbidden in the contracts of the great dairy and condensed milk companies. These are among the "things that everyone ought to know."

